# Supplementary material for: Mitochondrial Genome Variation in Polish Elite Athletes
Source: Int J Mol Sci. 2023 Aug 20;24(16):12992. doi: 10.3390/ijms241612992 (PMC10454803; doi:10.3390/ijms241612992)
Supplement: Supplementary file 1 [file ijms-24-12992-s001.zip › Supplementary Materials S2.pdf]

Determination of mtDNA copy number – detailed description:

The mitochondrial target gene was *MT-ND1* (GenBank reference sequence NC_012920) and the nuclear target gene was *B2M* (GenBank reference sequence NG_012920). The primers were as follows: *MT-ND1* forward primer 5'-TCGACCTTGCCGAAGGG-3' (position in reference sequence: m.3899-3915) and reverse primer 5'-TGTTTGTGTATTCGGCTATGAA-3' (m. 3997-3976) with product length of 99 bp; *B2M* forward primer 5'-CAGGTACTCCAAAGATTCAGG-3' (position in reference sequence: g.8944-8964) and reverse primer 5'-GTCAACTTCAATGTCGGATGG-3' (g.9053-9033) with product length of 110 bp. Reactions were performed in triplicate in 10 µl volumes on 384-well plates using LightCycler 480 SYBR Green I Master mix (Roche) following manufacturer's procedure with final concentration of primers for *MT-ND1* 0.8 µM and for *B2M* - 0.5 µM. Template DNA was added to each well in 2 µl. To ensure more accuracy and that samples will be detected within an optimal detection range for both genes, two template amounts were used for both reactions: 2 ng and 0.2 ng. Thermal cycling conditions followed the manufacturer's protocol LightCycler 480 Multiwell Plate 384: 45 cycles of denaturation at 95° for 10 s, primer annealing at 56 °C for 10 s, and primer extension at 72 °C for 10 s. Melting curve analysis was performed in each PCR run. An absolute quantification of the mtDNA copy number was performed using standard curves obtained for eight serial tenfold dilutions of pRS426 plasmid with cloned single copies of the *MT-ND1* and *B2M* gene fragments analyzed in the real-time PCR assay and of known (calculated) concentration, that is copy number in specified volume, ranging from 10^8 to 10^1 *MT-ND1* and *B2M* copies in each well. The PCR assay for the mitochondrial and nuclear genes were performed in parallel on the same plate together with the standard curves included in every run to minimize plate-to-plate variance error. An synthetic 498 bp oligonucleotide containing a single copy of the *MT-ND1* and *B2M* gene fragments analyzed in the real-time PCR assay was used as a calibrator for the both assays on each plate. This was done to control for the inter- and intra-experiment variance, as the *MT-ND1* and *B2M* copy number ratio for the calibrator sample should always equal 1. The mtDNA copy number was normalized per haploid genome and calculated as the *MT-ND1*/*B2M* ratio for each subject (the results for the triplicates for the both template DNA inputs were averaged).

Table S1 Comparison of the number of individuals with 0, 1, 2 or more rare (≤0.5% of population frequency), heteroplasmic, non-synonymous and synonymous mtDNA variants among sportsmen and control individuals.

**Number of rare mtDNA variants**	**Power athletes (n=52)**	**Endurance athletes (n=47)**	**Control group (n=97)**	***p*-value***
**0**	3	8	9	P+E vs C p=0.17 P vs C p=0.27 E vs C p=0.26 P vs E p=0.76
**1**	12	9	15	
**2**	14	10	16	
**3**	6	4	22	
**4**	11	6	13	
**5**	2	2	10	
**6+**	4	8	12	
**Number of heteroplasmic mtDNA variants**	**Power athletes (n=52)**	**Endurance athletes (n=47)**	**Control group (n=97)**	***p*-value***
**0**	36	35	54	P+E vs C p=0.0079 P vs C p=0.07 E vs C p=0.01 P vs E p=0.51
**1**	13	11	28	
**2**	2	1	11	
**3**	1	0	3	
**4**	0	0	1	
**Number of non-synonymous mtDNA variants**	**Power athletes (n=52)**	**Endurance athletes (n=47)**	**Control group (n=97)**	***p*-value***
**0**	0	0	0	P+E vs C p=0.14 P vs C p=0.31 E vs C p=0.17 P vs E p=0.49
**1**	0	0	0	
**2**	5	14	12	
**3**	15	8	20	
**4**	11	2	9	
**5**	6	3	10	
**6**	2	6	17	
**7**	3	7	12	

**8**	5	5	10	
**9**	3	0	3	
**10+**	2	2	4	

**Number of synonymous mtDNA variants**	**Power athletes (n=52)**	**Endurance athletes (n=47)**	**Control group (n=97)**	***p*-value***
**0**	0	1	1	P+E vs C p=0.47 P vs C p=0.79 E vs C p=0.35 P vs E p=0.51
**1**	4	4	9	
**2**	6	10	9	
**3**	8	5	15	
**4**	3	2	3	
**5**	3	1	3	
**6**	2	3	8	
**7**	3	1	5	
**8**	3	1	2	
**9**	1	2	2	
**10**	0	1	5	
**11**	6	5	8	
**12**	4	3	7	
**13**	5	3	7	
**14**	1	1	4	
**15+**	3	4	9	

*Mann-Whitney test

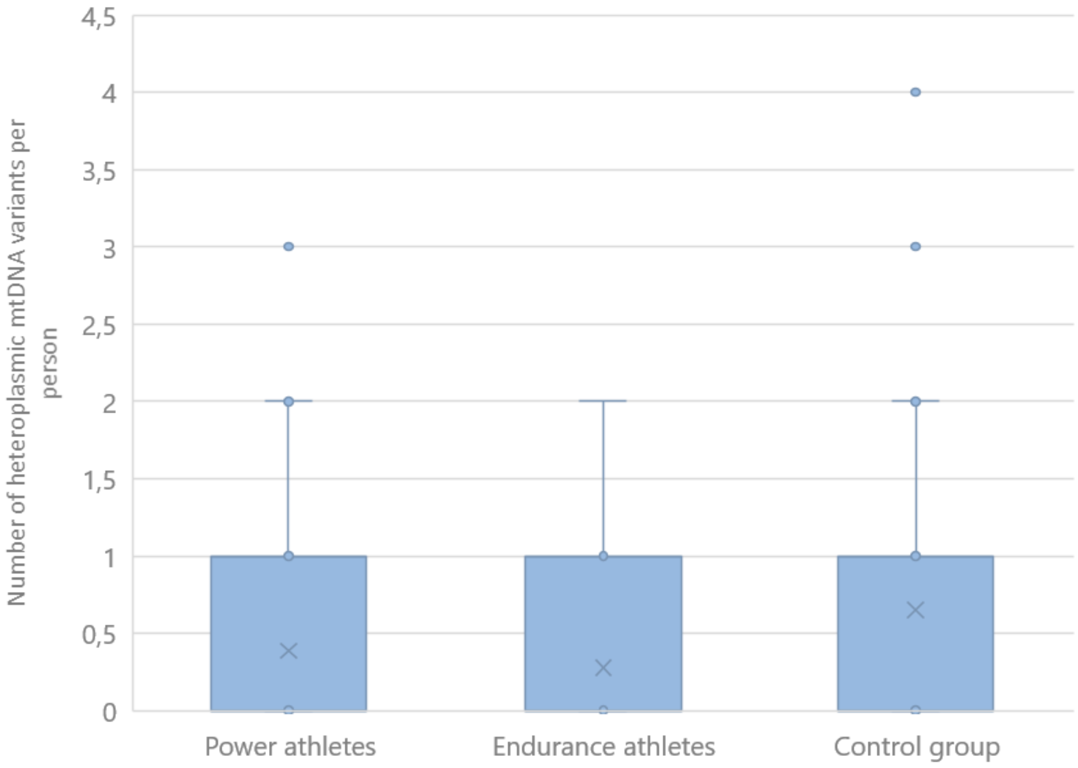

Figure S1. Distribution of heteroplasmic mtDNA variants in athletes and controls. Mean values are marked with a cross (all athletes 0.33 ± 0.59, power athletes 0.38 ± 0.66, endurance athletes 0.28 ± 0.50, controls 0.65 ± 0.88, mean ± SD). Endurance athletes, but not power athletes, had significantly less heteroplasmic mtDNA variants than controls (P+E vs C p=0.0079, P vs C p=0.07, E vs C p=0.01, P vs E p=0.51, Mann-Whitney test).
